# Validity testing of the Research Competence Scale for special education school teachers: an empirical study from China

**DOI:** 10.3389/fpsyg.2026.1863299

**Published:** 2026-08-25

**Authors:** Tong-Yao Jin, Xinchuang Shi, Rongdi Chen

**Affiliations:** 1 Suan Sunandha Rajabhat University, Bangkok, Thailand; 2 Zhengzhou Tourism College, Zhengzhou, China; 3 Zhejiang Provincial Special Education Guidance Center, Hangzhou, China

**Keywords:** China, psychometric validation, Research Competence, scale development, special education teachers

## Abstract

**Inroduction:**

Research competence (RC) is increasingly recognized as an essential component of teacher professionalism, particularly in special education where evidence-informed decision-making is critical. However, measurement instruments specifically designed to assess RC among frontline special education teachers remain limited. This study aimed to culturally adapt and validate the Research Competence Scale (RCS) within the context of Chinese special education schools.

**Methods:**

A total of 807 valid responses were obtained from full-time special education teachers in Zhejiang Province, China. A preliminary 24-item version of the adapted RCS was first examined through exploratory factor analysis (EFA). After item refinement, a 23-item four-factor measurement model was further evaluated through confirmatory factor analysis (CFA). The adapted scale was also evaluated in terms of reliability, convergent validity, and discriminant validity.

**Results:**

The results supported a four-factor structure consisting of conceptual competency, methodological competency, data analysis and interpretation, and writing technical competency. The adapted scale demonstrated satisfactory reliability, convergent validity, and discriminant validity.

**Discussion:**

This study contributes to the literature by demonstrating that RC requires contextual adaptation when applied across different educational and cultural settings. The validated RCS provides a theoretically grounded and empirically supported instrument for understanding RC among Chinese special education teachers and may inform future research and professional development initiatives. Future studies should further examine the scale’s applicability across diverse regions and investigate its associations with evidence-based teaching practices and professional outcomes.

## 1 Introduction

In the context of the continuous development of higher education, RC has emerged as an indispensable core competency within the educational field (Sternberg, 1998; Gess et al., 2018; Ciraso-Calí et al., 2022). RC in higher education ensures that educators can effectively interpret scientific literature and research findings, conduct teaching activities proficiently, objectively and reliably assess student performance, and continuously enhance teaching quality (Gess et al., 2018). More broadly, RC encompasses the ability to apply knowledge and skills to address professional tasks within scientific contexts (Prosekov et al., 2020). In the realm of special education, where educators face increasingly complex challenges and diverse student needs, proficiency in teaching skills alone is insufficient; teachers must also possess robust RC to scientifically evaluate the effectiveness of teaching strategies (Brownell et al., 2008; Billingsley and Bettini, 2017; Amanonce et al., 2024). While RC have been widely recognized as essential in the professional development of general education teachers (Lunenberg and Willemse, 2006; Tatto, 2021; Wen and Zeng, 2025; Kowalczuk-Walędziak et al., 2025), empirical measurement studies in the field of special education remain notably limited.

In China’s special education practice, teachers must continuously adapt to non-standardized teaching situations. RC has emerged as a critical indicator of special education teachers’ professional competence, directly influencing teaching effectiveness and student learning outcomes (Leko et al., 2015; Billingsley and Bettini, 2017; Yerzhanova et al., 2025). From both theoretical and practical perspectives, RC not only enhances teachers’ capacity to select and implement appropriate teaching strategies but also deepens their understanding of special education practices (Al-khresheh, 2024). In essence, RC empowers special education teachers to transcend mere empiricism and embrace evidence-based practices. However, existing RC assessment instruments are predominantly confined to general higher education frameworks or Western cultural paradigms, which results in a lack of psychometric validation for specialized Chinese cohorts. Notably, the direct transplantation of these foreign scales—without accounting for the unique conceptual boundaries, structural teacher-student ratios, and policy-driven mandates of Chinese special schools—poses a significant risk of introducing construct irrelevance. This measurement gap leaves institutions without a psychometrically robust baseline to inform teacher training and national competency assessment standards.

To address the critical deficit of culturally validated instruments, this study operationalizes and psychometrically validates Insorio’s (2024) Research Competence (RC) framework within the context of Chinese special education. Crucially, RC is conceptualized as a behavioral and practice-embedded construct that is distinct from broader research literacy, which typically denotes the passive consumption of scientific knowledge. By accounting for the systemic realities of Chinese special education—particularly the administrative mandates and institutional career-advancement mechanisms that incentivize teacher inquiry—this study refines the conceptual boundaries of the original framework to capture the most salient behavioral indicators of Chinese educators. Through the establishment of a robust validation argument, this investigation not only yields a psychometrically sound tool for teacher self-assessment and professional development but also provides an empirical baseline for institutional evaluation and policymaking.

Rather than merely replicating validation, this study critically adapts Insorio’s (2024) framework to align with the unique institutional landscape of Chinese special education, operationalizing a refined structural model that captures the most salient professional competencies of frontline educators. Ultimately, the validation of this instrument serves three primary objectives: first, to equip teachers with a psychometric diagnostic tool for formative self-assessment; second, to establish a structured baseline for targeted continuous professional development; and third, to provide school administrators with an empirical foundation for data-driven institutional evaluation.

## 2 Literature review

### 2.1 Conceptual definition and boundaries of research competence

RC encompasses a range of specific abilities aimed at fostering multidimensional development in individuals at both cognitive and personality levels. These abilities include axiomatic cognition, personal growth, general education, social participation, practical work experience, educational literacy, cognitive skills, communication skills, and information processing (Sternberg, 1998). Historically, RC was defined as an individual’s ability to cope with non-standardized and uncertain situations (Van Der Vleuten, 1996). However, contemporary scholars increasingly view it as a comprehensive attribute essential for conducting high-quality research within a professional field (Nechypurenko et al., 2021; Caingcoy, 2020), emphasizing its professionalism and practice-oriented nature.

At the methodological level, RC is characterized by a structured, research-based approach to problem analysis, allowing individuals to conduct research activities centered around a specific theme or problem while adhering to systematic research steps (Lambrechts and Van Petegem, 2016). Moreover, RC represents a systematic, methodologically controllable, and transparent process, with its core focused on generating new knowledge through standardized research pathways (Thiel and Böttcher, 2014). This process is reflected not only in the application of operational skills but also in the complexity and comprehensiveness of the competence structure.

From a structural perspective, RC encompasses multiple attributes. Firstly, it exhibits an interdisciplinary orientation, grounded in a foundation of scientific and practical theories. Secondly, its implementation process is characterized by systematization and operationalization. Thirdly, it incorporates both knowledge and skills dimensions, reflecting the integrative nature of the concept of ability (Böttcher and Thiel, 2018). Consequently, RC is not merely a singular skill; rather, it is the result of the synergistic effect of multiple dimensions.

In educational contexts, RC is regarded as a crucial interdisciplinary skill within the education system, serving as a vital component of educational guidance, management, and training (Ciraso-Calí et al., 2022). Specifically, RC encompasses essential professional and scientific knowledge, teaching attitudes and values, as well as a variety of research skills and learning strategies, including information retrieval and screening, problem identification and definition, goal setting, data collection and analysis, and result interpretation and presentation. Moreover, this competence extends to teamwork, academic writing, creativity in educational settings, and communication skills (Gómez-Núñez et al., 2023).

To establish a rigorous measurement model, RC as a latent construct must be clearly distinguished from its closely related conceptual neighbors, such as research literacy, research self-efficacy, and research engagement. While research literacy primarily emphasizes an individual’s static knowledge base and conceptual understanding of science, and research self-efficacy assesses one’s subjective psychological beliefs regarding their RC, RC specifically focuses on the behavioral, practice-oriented, and dynamic application of the operational skills required to systematically execute a research project. Furthermore, distinct from research engagement, which represents the frequency or degree of participation in research activities, RC reflects the underlying professional capacity and methodological rigor necessary to generate valid new knowledge.

### 2.2 Theoretical foundations of research competence

In teacher education, RC is widely regarded as one of the core competencies that teacher trainees must possess. Its development not only supports student learning but also promotes the improvement of educational quality (Thiem et al., 2023). Further research indicates that teacher education should systematically cultivate RC to enhance both teachers’ professional practice and teaching quality (Tatto, 2021). Consequently, RC is defined as a multifaceted and complex construct that encompasses the knowledge, skills, attitudes, and behaviors essential for effective research practice. This includes critical thinking, self-directed learning, and the capacity to organize and implement research activities (Matjašič and Vogrinc, 2024). Moreover, in the realm of educational research, RC signifies not only the accumulation of knowledge but also a profound understanding and effective management of the research process, rendering it an indispensable core quality for educational researchers (Tynjälä et al., 1997).

From a theoretical perspective, the formation of RC is rooted in multiple learning and cognitive theories. First, metacognitive theory emphasizes an individual’s ability to monitor and regulate their cognitive processes (Flavell, 1979). This ability manifests in the research process as reflection on research design, evaluation of evidence validity, and dynamic adjustment of research strategies. Second, scientific inquiry theory, based on Dewey’s (1938) philosophy of science, posits that RC is a systematic, evidence-based knowledge construction process, encompassing key stages such as hypothesis formulation, research design, data collection and analysis, and conclusion verification. This indicates that RC fundamentally involves the ability to construct knowledge using scientific methods. Finally, constructivist learning theory, from the perspective of social interaction and experiential construction, argues that RC is gradually formed through practical engagement and knowledge construction (Greeno, 1998), and is continuously developed through participation in the academic community and reflective practice (Rees et al., 2007). Furthermore, this theory highlights that RC improves progressively through practice, feedback, and collaborative learning, exhibiting distinct contextual and developmental characteristics (Nyikos and Hashimoto, 1997).

RC is a multi-dimensional, interdisciplinary, and developable comprehensive skill set. It is characterized by an individual’s capacity to integrate knowledge, skills, and attitudes within a specific field to conduct research activities systematically and with methodological rigor. This ability enables the analysis of problems and the generation of new knowledge.

### 2.3 Research competence in the context of special education

The development of teachers’ RC is grounded in multiple theoretical frameworks, including metacognitive theory, scientific inquiry theory, and constructivist learning theory. In the realm of special education, teachers must engage in continuous reflection on their teaching strategies and methods to effectively address student behavior. Given the inherent complexity and variability of special education, exceptional educators must possess independent RC to scientifically assess the applicability of teaching materials and intervention tools (Brownell et al., 2010; Byrd and Alexander, 2020; Amanonce et al., 2024). This model of research-based teaching practice not only aids teachers in selecting the most suitable strategies but also enhances their understanding of special education theory (Brownell et al., 2008; Özdemir and Kılıç, 2023). Particularly in educational settings characterized by uneven resource allocation and substantial teacher workloads, educators are required to employ scientific thinking methods, continuously refining their teaching through observation, hypothesis formulation, experimentation, and reflection (Liwanag et al., 2023; Rodríguez-Dorta and Borges, 2017). Consequently, RC transcends being merely an academic skill within the domain of special education; it serves as a fundamental driving force to ensure that disadvantaged students receive high-quality and scientifically-informed educational services.

Unlike university-based researchers, special education teachers primarily engage in practice-oriented inquiry rather than formal academic knowledge production. Their research activities are typically embedded within daily instructional decision-making processes, encompassing student assessment, individualized intervention planning, curriculum adaptation, and evaluation of educational strategies. Consequently, RC among special education teachers represents an applied professional capability that empowers educators to systematically identify instructional problems, collect and interpret evidence, and make data-informed decisions aimed at improving educational outcomes.

This practice-oriented nature suggests that the dimensional structure of teachers’ RC may differ from traditional RC models developed for academic researchers or teacher candidates. While academic-oriented research frameworks often emphasize scholarly communication, knowledge dissemination, and research motivation as critical components, the RC of special education teachers may place greater emphasis on methodological execution, evidence interpretation, and the practical application of research processes. Consequently, it is necessary to develop a context-sensitive measurement framework to accurately capture the unique competency requirements of special education teachers engaged in evidence-based educational practice.

### 2.4 Contextual refinement of the research competence framework and hypothesized structure

Insorio’s (2024) original framework conceptualizes teacher RC through seven latent dimensions: Conceptual, Methodological, Data Analysis and Interpretation, Technological, Dissemination and Utilization, Extrinsic Motivation and Intrinsic Motivation. This framework provides a comprehensive conceptualization of RC by integrating both capability-related and psychological dimensions. However, its direct application to Chinese special education teachers requires careful contextual adaptation because the professional roles, research purposes, and practical demands of special education teachers differ from those of academic researchers or teacher candidates.

Specifically, RC among special education teachers is primarily embedded within evidence-based instructional practice rather than scholarly knowledge production. Therefore, the present study adopts a competence-centered perspective, focusing on the observable knowledge, skills, and operational capabilities required for conducting practice-oriented inquiry. Based on this conceptual boundary, dimensions representing psychological dispositions and research outcomes were reconsidered to avoid construct overlap and improve measurement clarity.

The motivational dimensions (Intrinsic Motivation and Extrinsic Motivation) were excluded because they represent psychological antecedents that influence research engagement rather than the competence itself. Although motivation may facilitate the development and application of RC, it reflects individuals’ willingness or intention to engage in research activities rather than their actual capability to perform research tasks. Maintaining this conceptual distinction is consistent with competency-based measurement approaches that separate underlying motivational attributes from demonstrated abilities.

Furthermore, the Communication and Utilization dimension was removed due to contextual considerations. In academic research contexts, communication and utilization refer to activities such as scholarly dissemination, publication, and knowledge transfer. However, for special education teachers, research activities are primarily oriented toward solving immediate educational problems, including student assessment, intervention adjustment, and instructional improvement. Therefore, communication and utilization represent potential consequences or applications of RC rather than core competency components required to conduct practice-based inquiry.

In contrast, the technological dimension was retained because digital technologies increasingly function as essential tools throughout the research process, including information retrieval, data management, analytical procedures, and evidence-based decision-making. Within contemporary special education practice, technological competence represents an enabling capability that supports teachers’ engagement in systematic inquiry. Accordingly, rather than directly transferring an existing framework, this study seeks to establish a context-sensitive conceptualization of RC that reflects the practice-oriented inquiry demands of Chinese special education teachers.

## 3 Methods

### 3.1 Participants and sampling procedure

This study focused on full-time teachers in special education schools located in Zhejiang Province, China. According to official statistics, Zhejiang Province encompasses a land area of 105,500 square kilometers, comprises 11 prefecture-level cities, hosts 90 special education schools, and employs approximately 3,200 full-time special education teachers. Given the geographically dispersed nature of these schools and the practical challenges associated with reaching the target population, this study employed a two-stage cluster sampling method. Schools served as the primary sampling unit, while full-time teachers from the participating schools constituted the survey subjects. All eligible full-time teachers from these schools were invited to participate voluntarily in the study.

This study included 35 special education schools located in various prefecture-level cities across Zhejiang Province. These schools encompass diverse geographical landscapes and educational systems, including metropolitan areas, coastal regions, mountainous areas, island regions, and regions inhabited by ethnic minorities. Furthermore, the participating schools exhibit differences in their institutional characteristics and the types of students with special needs, reflecting the diversity within Zhejiang Province’s special education system. Although this study did not employ probability sampling to achieve statistical representativeness, the diverse sample coverage still enables us to capture the common characteristics and differentiated needs of special education teachers in Zhejiang Province across different developmental environments, thus providing a foundation for the generalization of subsequent research findings.

This study included 35 special education schools located in various prefecture-level cities across Zhejiang Province, which encompass diverse geographical landscapes and educational systems, including metropolitan areas, coastal regions, mountainous areas, island regions, and regions inhabited by ethnic minorities. Furthermore, the participating schools exhibit differences in their institutional characteristics and the types of students with special needs, reflecting the diversity within Zhejiang Province’s special education system. Although this study did not employ probability sampling to achieve statistical representativeness, the diverse sample coverage still enables us to capture the common characteristics and differentiated needs of special education teachers in Zhejiang Province across different developmental environments, thereby providing a foundation for the generalization of subsequent research findings.

To establish an independent validation framework, data collection was conducted in two phases. In the first phase, with the assistance of the local special education resource center, questionnaires were distributed to full-time teachers at participating schools, resulting in 300 valid questionnaires. After data screening, which included the removal of invalid responses characterized by uniform or linear response patterns, 257 valid responses were retained, yielding an effectiveness rate of 85.66%. This sample served as the calibration sample for exploratory factor analysis (EFA).

In the second phase, samples were collected from the same participating schools. To prevent sample overlap, teachers who participated in the first phase were excluded, and other eligible full-time teachers were invited to complete the questionnaire. A total of 583 questionnaires were collected, and after data screening, 550 valid responses were retained, resulting in a validity rate of 94.34%. This independent sample was utilized for confirmatory factor analysis (CFA) and subsequent assessments of reliability and validity.

### 3.2 Instrument adaptation and development

#### 3.2.1 Original instrument and conceptual adaptation

The Research Competence Scale (RCS) is adapted from the Teacher RC Framework developed by Insorio (2024). The original tool conceptualizes teacher RC as a multidimensional structure comprising seven dimensions: Conceptual, Methodological, Data Analysis and Interpretation, Technological, Dissemination and Utilization, Extrinsic Motivation and Intrinsic Motivation. The original Questionnaire contains 70 items, with 10 items for each dimension.

To enhance the applicability of this tool for special education teachers in China, this study employed a contextual adaptation approach rather than directly replicating the original framework. Based on theoretical considerations and the practical characteristics of research activities among frontline special education teachers, the study excluded three dimensions: intrinsic motivation, extrinsic motivation, and communication and application skills. These dimensions were conceptually regarded as distinct from research skills, or relatively distant from the daily inquiry practices of special education teachers in schools. Therefore, the revised framework encompasses four primary capability dimensions: Conceptual competence, Methodological competence, Data analysis and Interpretation, and Writing technical competency.

#### 3.2.2 Translation and cultural adaptation

To ensure linguistic accuracy and cross-cultural applicability, the translation and debugging process adhered to established recommendations for translation and back-translation in the context of cross-cultural scale development. Initially, two bilingual researchers with expertise in special education independently translated the original English version into Chinese. Throughout the translation process, particular emphasis was placed on the precision of terminology, the fluency of expression, and the relevance to the Chinese special education context.

Subsequently, the two translations were compared and integrated to create a preliminary Chinese version. An independent bilingual expert, not involved in the initial translation process, then translated this Chinese version back into English. The back-translated version was systematically compared with the original text item by item to identify potential semantic discrepancies and ensure conceptual equivalence.

Upon completion of the translation, the research team conducted an initial screening of the items. Items deemed irrelevant to the research practices of Chinese special education teachers, culturally inappropriate, or inconsistent with the adapted conceptual framework were revised. As a result of these modifications, the initial item bank was reduced from 70 items to a preliminary version containing 50 items. All items were assessed using a five-point Likert scale, with responses ranging from 1 (strongly disagree) to 5 (strongly agree).

#### 3.2.3 Content validation and expert review

To ensure content validity and assess its contextual applicability, this study employed a two-stage expert review process, inviting frontline teachers and academic experts to participate.

In the first stage, we invited five experienced special education teachers to evaluate the initial Chinese draft of the RCS. All experts possessed over 10 years of frontline teaching experience and had participated in municipal-level special education research projects or teacher training activities. Through semi-structured interviews and written feedback, the experts assessed each item based on clarity, relevance, and alignment with the professional context of Chinese special education teachers.

Based on the teachers’ feedback, we made the following revisions: (1) eliminated duplicate or semantically overlapping items to enhance conceptual clarity; (2) revised vague expressions containing implicit assumptions; and (3) adjusted culturally specific expressions to better align with the research environment of Chinese special education schools. In the second stage, we invited five academic experts from higher education institutions specializing in special education and educational management to conduct a further evaluation. These experts possessed between 10 to 35 years of research experience in special education teacher education and special education management. They reviewed each entry based on three aspects: semantic equivalence, cultural appropriateness, and content coverage.

After multiple rounds of discussion and refinement, the expert panel reached a consensus regarding the clarity, cultural relevance, and theoretical consistency of the revised tool. The final preliminary version, which comprises 24 items, was determined to possess satisfactory content validity and is suitable for assessing the RC of special education teachers in China.

### 3.3 Data collection procedure

Data collection for this study was facilitated by the regional professional support network established by the Zhejiang Provincial Special Education Resource Center. It is important to clarify that this resource center primarily functions as a platform for professional development, technical support, and regional collaboration, rather than as an administrative entity. Consequently, school participation was based on open invitations and voluntary engagement, rather than obligatory administrative mandates. Specifically, the research team initially communicated with the Zhejiang Provincial Special Education Resource Center, which subsequently disseminated the research invitation and survey instructions to the municipal special education resource centers. Following this, the municipal resource centers assisted in distributing the questionnaire links to relevant personnel in participating schools within their jurisdictions, and the schools organized eligible full-time teachers to voluntarily complete the questionnaires.

The questionnaire was administered online, with the survey link disseminated via internal communication platforms frequently utilized by Chinese educators, such as WeChat groups and QQ groups. Upon accessing the questionnaire page, participants were required to review the research instructions, which included the research objectives, methods of participation, principles of anonymity, and measures for data confidentiality. Participants could proceed to complete the questionnaire only after providing their informed consent. Participation was entirely voluntary, and no personally identifiable information was gathered during the research process.

To ensure data quality, this study implemented several rigorous quality control procedures. First, the electronic questionnaire system automatically recorded the completion status of each questionnaire, eliminating incomplete or evidently invalid submissions. Second, the consistency of responses was assessed, resulting in the removal of straight-line responses (where all items were answered identically) and samples exhibiting clear randomness or carelessness. Following the data cleaning process, only valid questionnaires that met the established quality criteria were retained for subsequent statistical analysis.

This research protocol underwent ethical review by the researchers’ institutions prior to data collection and adhered to the relevant ethical principles outlined in the Declaration of Helsinki. The two phases of data collection were conducted sequentially, with a 30-day interval between them. To ensure the independence of the calibration and validation samples, the online survey system was configured to prevent researchers who had completed the first phase from participating in the second phase of data collection.

### 3.4 Data analysis

This study utilized SPSS 26.0 and AMOS 24.0 software for data analysis. To systematically examine the structural characteristics and psychometric properties of the adapted RCS, a phased analysis strategy was employed. Initially, exploratory factor analysis (EFA) was performed using the calibration samples collected in the first phase to investigate the potential factor structure of the scale. Subsequently, confirmatory factor analysis (CFA) was conducted on independent samples in the second phase to assess the stability of the proposed theoretical model. Furthermore, this study comprehensively evaluated the reliability and validity of the scale through analyses of internal consistency reliability, convergent validity, discriminant validity, and measurement invariance.

#### 3.4.1 Exploratory factor analysis (EFA)

In the initial phase of this study (*N* = 257), exploratory factor analysis was employed to investigate the underlying structure of the adapted RCS. Prior to conducting the factor analysis, the appropriateness of the data for such analysis was evaluated using the Kaiser–Meyer–Olkin (KMO) test and Bartlett’s test of sphericity. The KMO value was utilized to assess the adequacy of the correlations among the variables, while Bartlett’s test of sphericity was implemented to determine whether the correlation matrix significantly deviated from the identity matrix.

In the factor extraction process, this study employed principal axis factoring (PAF) to extract latent factors and utilized varimax rotation to enhance the interpretability of the factor structure (Fabrigar et al., 1999). The determination of the number of factors was based on a comprehensive consideration of eigenvalues, scree plots, and theoretical interpretability, rather than solely relying on the criterion of eigenvalues greater than 1.

During the project screening process, factor loadings and cross-loadings were comprehensively evaluated. Items with factor loadings below 0.50, as well as items exhibiting substantial cross-loadings across multiple factors or inadequate item discrimination, were considered for removal based on established EFA criteria(Hair et al., 2019; Costello and Osborne, 2005). Through this analytical process, the potential factor structure of the adapted RCS was ultimately determined, establishing a foundation for subsequent confirmatory factor analysis.

#### 3.4.2 Confirmatory factor analysis (CFA)

In the second phase involving independent samples (*N* = 550), confirmatory factor analysis (CFA) was conducted to validate the factor structure identified through exploratory factor analysis. CFA was executed using AMOS 24.0 software to evaluate the goodness of fit between the theoretically proposed model and the actual data.

The model fit was evaluated using several indicators, including the chi-squared degrees of freedom ratio (*χ*^2^/df), the Comparative Fit Index (CFI), the Tucker–Lewis Index (TLI), the Root Mean Square Error of Approximation (RMSEA), and the Standardized Root Mean Square Residual (SRMR). This study conducted a comprehensive assessment of the model’s structural fit through these multiple indicators, further determining whether the adapted four-factor structure could more effectively elucidate the underlying structure of special education teachers’ RC.

#### 3.4.3 Reliability and validity assessment

To further evaluate the psychometric quality of the adapted RCS, this study examined its internal consistency reliability, convergent validity, and discriminant validity.

First, Cronbach’s alpha coefficient and composite reliability (CR) were employed to assess the internal consistency of the scale and its dimensions. Generally, an alpha coefficient and CR value exceeding 0.70 indicate satisfactory internal consistency reliability.

Second, standardized factor loadings and average variance extracted (AVE) were utilized to evaluate convergent validity. Factor loadings above 0.50 and AVE values exceeding 0.50 are typically regarded as indicative of the latent variables effectively explaining the common variance of their measurement items.

Finally, the Fornell–Larcker criterion and the heterotrait–monotrait ratio (HTMT) were applied to assess the discriminant validity among the latent variables. Strong discriminant validity among the latent variables suggests that different dimensions of RC represent distinct yet related theoretical constructs.

## 4 Results

### 4.1 Demographic characteristics

In the initial phase of this study, questionnaires were distributed to special education resource centers across various cities and prefectures in Zhejiang Province, utilizing the resources of the Zhejiang Provincial Special Education Resource Center. A total of 300 valid questionnaires were collected during this phase. After thorough data screening, no data was found to be missing, and invalid samples exhibiting linear response patterns were eliminated, resulting in 257 valid samples and a sample recovery rate of approximately 85.66%. The demographic composition of the sample was predominantly female, with a significant proportion of middle-aged teachers, particularly those aged 40–49. In terms of educational qualifications, the majority of participants possessed a bachelor’s degree, followed by those with associate degrees, while the number of teachers holding postgraduate degrees was relatively limited. The distribution of teaching experience was fairly balanced, showcasing a considerable number of both junior and senior teachers. Overall, this sample accurately reflects the structural characteristics of in-service special education teachers, with a notable emphasis on experienced practitioners.

In the second phase, A total of 583 valid questionnaires were collected in this phase. Following data screening, no omissions were detected. After excluding invalid samples characterized by linear responses, 550 valid samples were retained, resulting in a sample recovery rate of approximately 94.34%. The sample in the second phase maintained a stable gender structure but exhibited a significant increase in the proportion of younger and less experienced teachers. The 20–29 age group constituted the highest proportion of participants, indicating a predominance of early-career teachers in this sample. Consistent with the first phase, most respondents possessed a bachelor’s degree; however, there was a slight increase in the proportion of those holding an associate’s degree. The distribution of teaching experience was skewed towards teachers with less than 6 years of experience.

The demographic characteristics of the two-stage samples indicate a relatively stable gender structure, predominantly consisting of female teachers. Regarding educational background, the samples mainly include teachers with bachelor’s and associate’s degrees, while the proportion of postgraduate degree holders is relatively low, and there are no doctoral degree holders. This sample group aligns with the structure of the special education teacher workforce as reported by the Zhejiang Provincial Education Administration. Table 1 summarizes the overall sample characteristics.

**Table 1:** Population sample.

Variable	Category	The first phase		The second phase	
		*N*	%	*N*	%
Gender	Male	40	15.6	92	16.7
	Female	217	84.4	458	83.3
Age	20–29 years old	38	14.8	235	42.8
	30–39 years old	58	22.6	164	29.8
	40–49 years old	93	36.2	100	18.2
	Above 49 years old	51	19.8	51	9.2
The highest education	Associate degree	93	36.2	226	41.1
	Bachelor’s degree	137	53.3	275	50.0
	Master’s degree	27	10.5	49	8.9
	Doctoral degree	0	0	0	0
Work experience	Under 6 years	76	29.6	189	34.4
	6–10 years	71	27.6	136	24.8
	11–15 years	46	17.9	80	14.55
	16–20 years	10	3.9	37	6.7
	Above 20 years	54	21.0	108	19.6
	Total	257	100.00	550	100.00

### 4.2 Exploratory factor analysis (EFA)

Before conducting exploratory factor analysis (EFA), the suitability of the data for factor extraction was assessed using the Kaiser–Meyer–Olkin (KMO) measure and Bartlett’s test of sphericity. The results demonstrated that the data were appropriate for factor analysis, with a KMO value of 0.952 and a significant result from Bartlett’s test of sphericity (*χ*^2^ = 7134.442, df = 276, *p* < 0.001). Following this, exploratory factor analysis was performed using the principal axis factoring (PAF) method with Promax rotation. The Promax rotation method was chosen because the extracted factors were theoretically anticipated to be correlated rather than completely independent. The rotation procedure successfully converged after seven iterations (See Table 2).

**Table 2:** EFA results.

Pattern matrix^a^	Component			
	DAI	CC	WTC	MC
17. Interpret data generated by data analysis software.	**0.890**	0.067	−0.024	−0.084
19. Draw valid conclusions based on the data provided.	**0.856**	0.011	0.093	−0.049
18. Apply theoretical frameworks to interpret the data.	**0.837**	0.072	0.077	−0.073
16. Interpret data based on literature support.	**0.812**	0.008	0.083	0.025
14. Compare research findings with prior/existing studies.	**0.806**	0.013	0.020	0.093
13. Present collected data in narrative, tabular, or graphical form.	**0.742**	−0.010	−0.013	0.190
20. Formulate appropriate recommendations based on the conclusions.	**0.740**	0.015	0.129	0.067
15. Propose the potential implications of the research.	**0.728**	0.031	0.034	0.134
12. Identify appropriate statistical techniques for data analysis.	**0.536**	0.004	−0.097	**0.460**
4. Identify appropriate data collection instruments.	0.112	**0.997**	−0.080	−0.134
3. Determine the research design, sampling methods, and sample size.	0.110	**0.953**	−0.134	−0.086
2. State the research objectives and research questions clearly.	0.005	**0.880**	0.019	−0.045
1. School (classroom) problems can be transformed into viable research questions.	−0.080	**0.840**	0.072	−0.023
6. Present and analyze data correctly	0.029	**0.655**	0.056	0.205
5. Select an appropriate data analysis tool	0.062	**0.653**	0.001	0.216
24. Carefully compose each section of the research paper.	−0.022	−0.090	**0.895**	0.114
22. Demonstrate familiarity with standard research paper format.	0.032	−0.002	**0.876**	0.042
23. Incorporate citations from credible sources when discussing or summarizing literature.	0.090	0.021	**0.874**	−0.128
21. Use formal academic language to demonstrate scholarly writing.	0.147	0.021	**0.804**	−0.101
11. Manage and collect data ethically	0.295	−0.170	**−0**.071	**0.801**
9. Communicate effectively with researchers and collaborators.	−0.018	0.171	0.005	**0.760**
10. Obtain informed consent from participants and their parents/guardians.	0.200	−0.041	−0.079	**0.734**
7. Use online resources to find relevant literature.	−0.157	0.357	0.144	**0.573**
8. Implement innovative intervention strategies according to the research plan.	−0.105	0.398	0.101	**0.534**
Eigenvalue	14.317	2.568	1.405	0.992
Explained variance (%)	59.654	10.702	5.856	4.132
Total variance (%)	59.654	70.356	76.212	80.345

Data analysis and interpretation (DAI), conceptual competency (CC), writing technical competency (WTC), methodological competency (MC).

Extraction method: principal axis factoring. rotation method: Promax with Kaiser normalization.

a Rotation converged in 7 iterations. Bold values indicate primary factor loadings (> 0.40).

The first factor, comprising 8 items (13–20), reflects the teacher’s ability to organize, analyze, and interpret research data, and to formulate reasonable judgments and practical suggestions based on research results; it retains its original designation, “data analysis and interpretation” (DAI).

The second factor, consisting of 6 items (1–6), involves research question identification, research design, sample selection, research tool determination, and data processing method selection, reflecting the methodological knowledge and skills required for teachers to conduct standardized research processes; thus, it is termed “conceptual competency” (CC).

The third factor, comprising 4 items (21–24), pertains to research paper writing, adherence to academic norms, literature citation, and the presentation of research results, reflecting the teacher’s ability to systematically organize and academically present research findings; therefore, it is labeled “writing technical competency” (WTC).

The fourth factor, consisting of five items (7–11), primarily concerns the utilization of literature resources, implementation of research interventions, research ethics, and the capacity to communicate effectively with research participants and relevant personnel; hence, it is designated “methodological competency” (MC).

Furthermore, item 12, “choosing the right data analysis tools,” exhibited significant cross-loading between the first factor (0.536) and the fourth factor (0.460), with a minimal difference between the two loadings, which could potentially affect factor discriminability; thus, it was removed according to the statistical criteria for scale revision. The removal of this item resulted in a clearer scale structure, with each factor demonstrating substantial theoretical explanatory power. Overall, the EFA results indicate that the adapted RCS (23 items) presents a situation-adaptive four-factor structure among special education teachers in China, providing empirical evidence for subsequent confirmatory factor analysis.

### 4.3 Reliability analysis

The internal consistency reliability of the adapted RCS was assessed using Cronbach ‘s alpha coefficients derived from the exploratory sample. According to Nunnally and Bernstein (1994), Cronbach ‘s alpha values exceeding 0.70 indicate acceptable reliability. As illustrated in Table 3, all four dimensions exhibited excellent internal consistency, with alpha coefficients ranging from 0.915 to 0.965. The corrected item-total correlation (CITC) values ranged from 0.775 to 0.902, surpassing the recommended threshold of 0.30, thereby suggesting that all retained items contributed sufficiently to their respective constructs.

**Table 3:** Reliability analysis of the Research Competence Scale.

Dimension	*n*	Cronbach’s *α*	CITC range
Data analysis and interpretation (DAI)	8	0.965	0.817–0.877
Conceptual competency (CC)	6	0.946	0.775–0.902
Writing technical competency (WTC)	4	0.939	0.786–0.815
Methodological competency (MC)	5	0.915	0.783–0.857

Specifically, the DAI dimension displayed the highest internal consistency reliability (*α* = 0.965), with CITC values ranging from 0.817 to 0.877. The CC dimension exhibited strong reliability (*α* = 0.946), with CITC values ranging from 0.775 to 0.902. The WTC dimension also demonstrated satisfactory internal consistency (*α* = 0.939), with CITC values ranging from 0.786 to 0.815.

Lastly, the MC dimension achieved an acceptable reliability coefficient (*α* = 0.915), with CITC values ranging from 0.783 to 0.857. All CITC values surpassed the commonly recommended threshold of 0.30, indicating that each item exhibited adequate consistency with its corresponding construct. Consequently, no items were excluded based on the reliability analysis, and the retained items were further subjected to exploratory factor analysis followed by confirmatory factor analysis.

### 4.4 Confirmatory factor analysis (CFA)

Confirmatory factor analysis (CFA) was conducted using AMOS 24.0 on the independent validation sample (*N* = 550) to assess the stability of the four-factor structure identified in the exploratory factor analysis. The measurement model comprised four correlated latent constructs: CC, MC, DAI, and WTC.

As shown in Table 4, the model fit indices indicated that the hypothesized four-factor model provided a highly satisfactory fit to the observed data. Although the chi-square statistic was statistically significant (*χ*^2^ = 607.763, df = 224), it is important to note the sensitivity of the chi-square to sample size; therefore, additional fit indices were examined. The ratio of chi-square to degrees of freedom was acceptable (*χ*^2^/df = 2.713). The absolute fit indices confirmed robust alignment, with GFI = 0.906, AGFI = 0.884, SRMR = 0.024, and RMSEA = 0.056 (recommended <0.08). Furthermore, the incremental fit indices demonstrated superior performance, with IFI = 0.970, TLI = 0.953, and CFI = 0.959, all comfortably exceeding the stringent benchmark of 0.90.

**Table 4:** Fit indices of the four-factor measurement model.

Fit index	Value	Recommended cutoff criteria
CMIN	607.763	—
DF	224	—
CMIN/DF	2.713	<3.00
GFI	0.906	≥0.90
AGFI	0.884	≥0.85
SRMR	0.024	<0.08
IFI	0.970	≥0.90
TLI	0.953	≥0.90
CFI	0.959	≥0.90
RMSEA	0.056	<0.08

Model fit criteria were adapted from Kline (2023), Hu and Bentler (1999), Wheaton et al. (1977), Jöreskog and Sörbom (1989), Browne and Cudeck (1993), Fornell and Larcker (1981).

**Figure 1:**
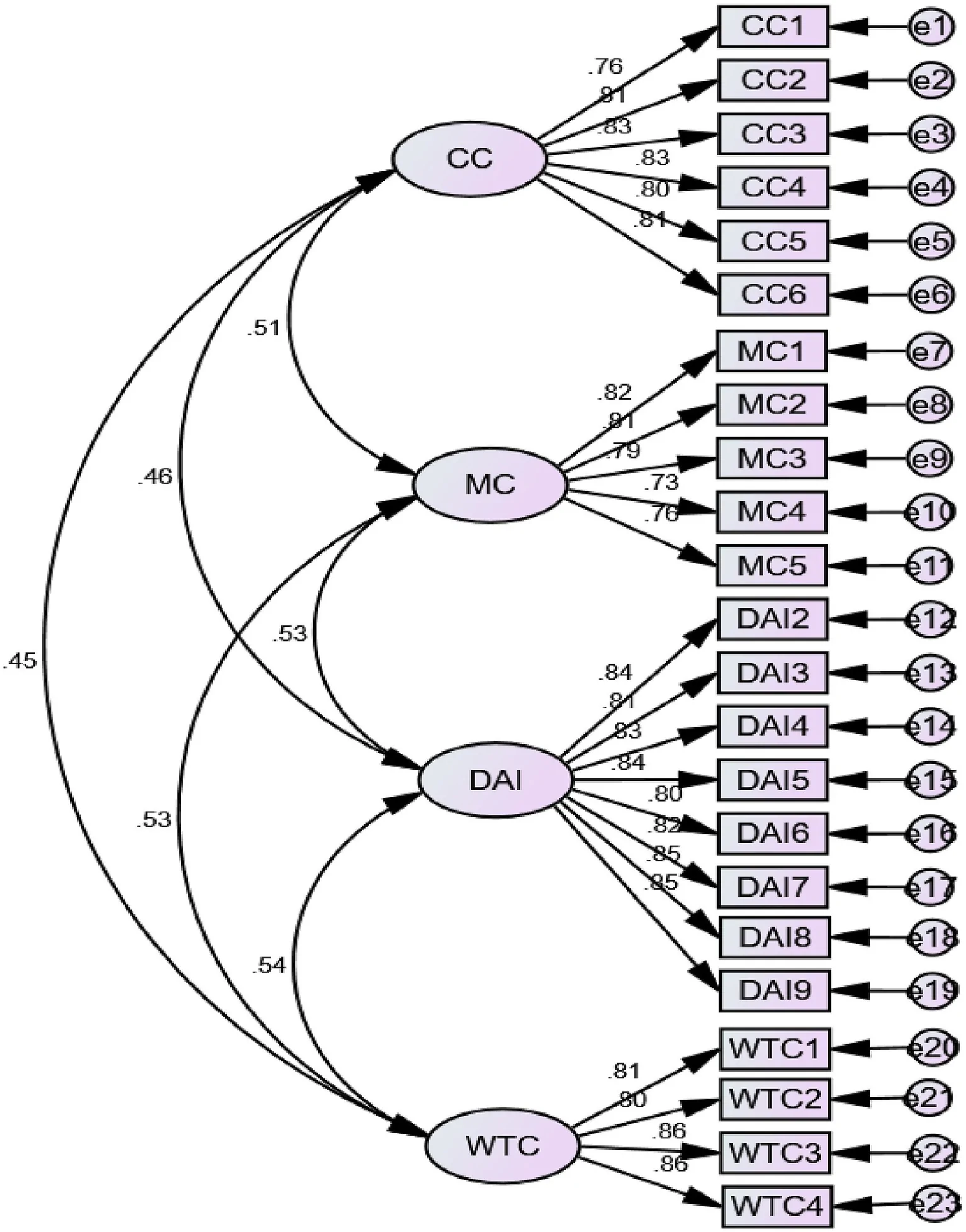
Standardized four-factor measurement model of the Research Competence Scale.

As illustrated in Figure 1, all standardized factor loadings were statistically significant and exceeded the recommended threshold of 0.50, ranging from 0.73 to 0.86, indicating satisfactory item reliability and convergent validity.

### 4.5 Convergent validity and discriminant validity

#### 4.5.1 Convergent validity

Convergent validity was evaluated by analyzing the standardized factor loadings, composite reliability (CR), and average variance extracted (AVE) of the four-factor measurement model. As shown in Table 5, all standardized factor loadings surpassed the recommended threshold of 0.70, with values ranging from 0.726 to 0.859, indicating that all observed items exhibited strong correlations with their respective latent constructs. The AVE values for the four dimensions ranged from 0.611 to 0.693, exceeding the recommended criterion of 0.50 proposed by Fornell and Larcker (1981). Specifically, the AVE values were 0.655 for CC, 0.611 for MC, 0.689 for DAI, and 0.693 for WTC. These findings suggest that each construct accounts for a significant proportion of variance in its corresponding measurement items. Moreover, the CR values ranged from 0.889 to 0.948, surpassing the recommended threshold of 0.70. These results indicate satisfactory internal consistency among the indicators within each construct. Collectively, the factor loadings, AVE, and CR results provide compelling evidence supporting the convergent validity of the adapted RCS.

**Table 5:** Composite reliability and convergent validity.

Variable	Item	Factor loading	AVE	CR
Conceptual competency (CC)	6	0.763–0.834	0.655	0.920
Methodological competency (MC)	5	0.726–0.820	0.611	0.889
Data analysis and interpretation (DAI)	8	0.795–0.854	0.689	0.948
Writing technical competency (WTC)	4	0.801–0.859	0.693	0.901

#### 4.5.2 Discriminant validity

Discriminant validity was assessed using the Fornell–Larcker criterion and the maximum shared variance (MSV). As illustrated in Table 6, the diagonal values represent the square roots of the AVE for each construct, while the off-diagonal values reflect the correlations between constructs. The square roots of AVE were 0.809 for CC, 0.782 for MC, 0.830 for DAI, and 0.832 for WTC.

**Table 6:** Discriminant validity of the Research Competence Scale.

Variable	CC	MC	DAI	WTC	MSV
Conceptual competency (CC)	**0.809**				0.261
Methodological competency (MC)	0.511^***^	**0.782**			0.284
Data analysis and interpretation (DAI)	0.458^***^	0.533^***^	**0.830**		0.295
Writing technical competency (WTC)	0.445^***^	0.528^***^	0.543^***^	**0.832**	0.295

Diagonal values represent the square roots of AVE, and off-diagonal values represent correlations among constructs. MSV, maximum shared variance. *p < 0.05, **p < 0.01, ***p < 0.001.

According to the Fornell–Larcker criterion, discriminant validity is affirmed when the square root of AVE for each construct exceeds its correlations with other constructs. The results demonstrate that the square roots of AVE consistently surpassed the inter-construct correlation coefficients. Specifically, the correlations among the four dimensions ranged from 0.445 to 0.543, whereas the corresponding square roots of AVE varied from 0.782 to 0.832.

Additionally, the MSV values ranged from 0.261 to 0.295, all of which were lower than their respective AVE values, thus further validating adequate discriminant validity. Consequently, the four dimensions of the RCS represent empirically distinct yet theoretically related constructs. These findings provide further support for the validity of the proposed four-factor measurement model.

## 5 Discussion

This study aimed to adapt and validate the RCS for full-time teachers working in special education schools in China. By refining the original framework proposed by Insorio (2024), we developed a context-sensitive four-factor model that includes CC, MC, DAI, and WTC. The findings provide empirical evidence supporting the reliability and validity of the adapted RCS, contributing to a more contextualized understanding of RC among frontline special education teachers.

### 5.1 Main findings and validation evidence

This study offers robust empirical evidence for the construct validity and psychometric properties of the adapted Chinese RCS for Special Education Teachers, thoroughly validating research hypotheses H1 and H2. By employing a sequential testing approach that integrates exploratory and confirmatory factor analyses, the findings demonstrate that the proposed four-factor model effectively captures the multidimensional structure of RC among special education teachers.

The results of the CFA unequivocally validated the stability of the four-factor structure. All model fit indices satisfied the optimal thresholds for psychometric evaluation, and each subscale displayed exceptional internal consistency along with distinct conceptual differentiation. This compellingly illustrates that the adapted scale, while preserving the independence of each dimension, effectively captures the RC of frontline special education teachers in key areas such as concepts, methods, data analysis, and technology application.

Compared to traditional Western scales, this study effectively eliminates construct redundancy while preserving the essential assessment dimensions. Although there exists a moderate correlation among the four competency dimensions, they function as complementary modules rather than interchangeable concepts. By closely aligning the item descriptions with the institutional context and practical needs of special education in China, this scale can more accurately diagnose the research bottlenecks faced by special education teachers. It thus serves as a reliable measurement tool for the subsequent assessment of the RC levels of special education teachers and the factors influencing them.

This study significantly enhances our localized understanding of teacher RC. In contrast to university researchers who focus on academic publications, frontline special education professionals primarily engage in practice-oriented inquiry. Their goal is to address everyday teaching challenges, assess the effectiveness of student behavioral interventions, and optimize IEPs (Wilmshurst and Brue, 2018; Sadler and Sugai, 2009). This finding robustly supports the perspective that “teacher RC is a complex, multidimensional construct that integrates cognitive understanding with evidence-driven practical application,” thereby providing highly ecologically valid empirical evidence for the advancement of the special education profession.

### 5.2 Theoretical rationale for contextual adaptation

To gain a deeper understanding of why the adapted four-factor model in this study demonstrates excellent structural fit and measurement stability, it is essential to systematically elaborate on the simplification of scale dimensions and localization adaptations from both theoretical and practical perspectives within the context of special education.

First, the adjustment to remove the Extrinsic Motivation and Intrinsic Motivation dimensions reflects a significant conceptual distinction between RC and motivational orientation. Motivation, whether arising from a teacher’s inherent interest in inquiry, a pursuit of professional growth, or influenced by external evaluations and incentive policies, can greatly affect the extent to which a teacher engages in research activities (Li and Xu, 2024; Hardré, 2012). However, motivation serves primarily as a psychological antecedent that drives behavior, rather than a direct manifestation or component of RC itself. Merging motivational orientation with competence constructs, which are grounded in actual knowledge, skills, and behaviors, obscures the theoretical boundaries between the two, thereby diminishing the scale’s clarity in measuring purely ‘competence’ constructs (Brown, 2023). Consequently, the deliberate exclusion of the motivational dimension enables the revised scale to more accurately and specifically evaluate the observable research knowledge and operational skills that teachers possess and utilize, thereby achieving a precise alignment between theoretical construction and measurement practice.

Secondly, the decision to exclude Dissemination and Utilization stems from a thorough consideration of the nature of research activities undertaken by special education teachers in China. Within the context of special education schools, teachers’ research efforts primarily concentrate on addressing immediate classroom teaching challenges, optimizing IEPs, and implementing behavioral interventions (Oliver and Reschly, 2010; Johnson and Semmelroth, 2014). In contrast to academic researchers in universities, frontline special education teachers possess fewer objective opportunities and job requirements to engage in extensive academic dissemination or systematic knowledge transformation. Consequently, this adjustment enhances the focus on abilities that are directly pertinent to the teachers’ daily research processes, emphasizing the assessment of core components of “research in action.” This includes how teachers identify teaching problems, monitor student behavior data, and modify intervention programs based on empirical evidence, rather than evaluating their capacity to publish academic papers or disseminate findings within academic networks. This emphasis does not diminish the importance of Dissemination and Utilization; rather, it recognizes that, in the current landscape of frontline special education practice in China, RC is primarily manifested as practical wisdom and actions embedded within daily work, aimed at directly fostering student development.

Third, refining the original framework does not diminish the standards for RC; rather, it reflects a contextualized reconstruction of the boundaries of this ability. This adjustment does not lower expectations for teachers’ capabilities. Instead, it offers a more precise and operational definition of the concept of ‘RC’ based on the specific context of frontline work in special education in China. The four-factor model integrates and strengthens dimensions closely linked to daily teaching—namely, conceptual ability, methodological ability, data analysis and interpretation ability, and technical ability—while temporarily setting aside systematic academic dissemination activities that are relatively marginalized in current frontline practice. This model highlights the core competencies required for special education teachers to conduct practice-oriented research. It moves away from the limitations of merely applying general models used by academic researchers, shifting towards the construction of a practice-oriented ability system rooted in special education classrooms and aimed at serving the development of children with special needs.

Consequently, this model provides a more culturally and professionally relevant conceptual framework for teacher RC. It aligns closely with the environment, resources, and work priorities of special education schools, thus effectively guiding and supporting special education teachers in developing and applying RC within their actual work context.

### 5.3 Practical implications for special education

The validated four-factor scale (RCS) developed in this study not only possesses psychometric value but also offers concrete practical pathways for teacher development and the enhancement of school-based research in special education institutions.

Firstly, it facilitates a more nuanced diagnosis of teachers’ RC. This scale departs from traditional evaluation methods that regard ‘RC’ as an abstract and generalized concept. Its empirically validated four-dimensional structure—CC, MC, DAI, and WTC—breaks down this construct into observable and measurable operational indicators. Consequently, school administrators can transcend vague impressions of teachers’ research competencies and accurately assess individual teacher profiles across specific dimensions. For instance, a teacher may excel at identifying pedagogical issues but struggle with the statistical analysis of student behavior data; alternatively, a team may demonstrate proficiency in utilizing technical tools while requiring enhancement in action research design. This refined diagnosis establishes a robust foundation for the subsequent development of personalized and precise support strategies.

Second, it drives precise and modular school-based professional development. The accurate “competency profiles” generated by the assessment provide direct guidance for school administrators and training coordinators to design intervention programs, moving away from the previous “one-size-fits-all” model of continuing education. For instance, for teachers who score lower in the methodology dimension, training can focus on the application of single-subject designs or observation and recording tools. In contrast, for teachers with limited data analysis capabilities, the training can emphasize evaluating the effectiveness of IEPs. This targeted approach, grounded in assessment data, effectively aligns limited training resources with teachers’ specific weaknesses, significantly enhancing the efficiency and effectiveness of professional development.

Third, it promotes collaborative inquiry and team building based on complementary abilities. The scale provides a universally standardized language for assessing RC, establishing a common cognitive foundation for schools to organize related activities and foster an inquiry-based atmosphere. By identifying individual teachers’ strengths and weaknesses, schools can systematically form research groups with complementary skills. For example, a special education teacher who excels at identifying classroom challenges and designing lessons can collaborate with a teacher skilled in data analysis or assistive technology to conduct action research aimed at addressing behavioral issues among children with special needs. This complementary collaboration can integrate individual strengths into collective wisdom, enhancing instructional decisions while cultivating an evidence-based, open, and collaborative inquiry culture at the school level.

Fourth, it promotes the transformation of special education teachers into ‘evidence-based reflective practitioners.’ This research highlights that RC is not merely an academic metric detached from classroom realities; instead, it constitutes a fundamental aspect of the professional competence of special education teachers. When these educators adeptly employ research tools to scrutinize their teaching practices, assess the effectiveness of interventions grounded in data evidence, and engage in systematic reflection on the intricate challenges of special education, research becomes truly internalized as a pivotal catalyst for refining teaching decisions. This process aids in bridging the divide between educational research and special education practice, encouraging teachers to evolve from experience-dependent instructors into reflective practitioners who can make informed decisions based on empirical evidence.

### 5.4 Limitations and future research directions

While this study promotes the localization of measurement of special education teachers’ RC, it also presents several limitations that must be carefully considered when interpreting and applying its conclusions. These limitations will also guide future research.

First, the geographical and cultural context of the sample is restricted. Although the sample includes special education teachers with varying types, teaching experience, and professional backgrounds within Zhejiang Province, which demonstrates good provincial representativeness, it remains confined to the developed coastal areas of eastern China. The levels of economic development, special education policy investment, and teacher training systems in Zhejiang Province may differ significantly from those in central and western regions. Consequently, the cross-sample universality and measurement invariance of this four-factor model across different regions and multicultural backgrounds nationwide require thorough examination. Future research should aim to expand the sampling coverage to a national level, investigate the applicability and equivalence of the scale across different regional education systems, and establish more robust external validity.

Second, the limitations of cross-sectional design in dynamic tracking are evident. This study employed a cross-sectional design, collecting data at a specific point in time. Consequently, it cannot reveal the dynamic evolution of teachers’ RC throughout their careers, nor can it assess the long-term effects of specific interventions. Future research should incorporate a longitudinal design, continuously measuring the same group at different time points (e.g., before and after special education teachers receive specialized training, or at various stages of their teaching experience). This approach will help construct a dynamic model of the development of special education teachers’ RC and explore the differentiated impacts of professional experience accumulation and institutional support on the growth of various dimensions of their abilities.

Third, social desirability bias and measurement bias in self-report scales present significant challenges. This study relied on teachers’ self-reports for data collection. While this method is convenient and reflects self-efficacy perceptions, it is susceptible to social desirability bias or conceptual misunderstandings, which may lead to the overestimation or underestimation of abilities. To construct a more comprehensive and objective assessment framework, future research should adopt a triangulation approach, actively incorporating multi-source data. For instance, by combining systematic observations of special education classrooms, quality assessments of IEPs, peer and school administration reviews, and objective school-based teaching research performance indicators, a holistic picture of teachers’ RC can be achieved.

Fourth, this study emphasizes the necessity for rigorous testing of criterion-related validity and predictive validity. It focuses on the localization adaptation of the scale, as well as the verification of construct validity and internal consistency, which are essential steps in the development of measurement tools. However, comprehensive validation of the tool necessitates an examination of its association with external standards and actual behavioral outcomes. Future research should delve deeper into the predictive relationship between RCS scale scores and teachers’ actual professional behaviors. For instance, a systematic investigation could be conducted to ascertain whether the RC scores of special education teachers can significantly predict the quality of their evidence-based teaching practices (EBP), the frequency of innovative teaching behaviors, and their tangible contributions to the individualized development of children with special needs. This would further enhance the scale’s practical value in policymaking and performance diagnosis.

## 6 Conclusion

This study investigates the localization and psychometric testing of the RCS for full-time teachers in Chinese special education schools. Building upon the original theoretical framework proposed by Insorio (2024), the study systematically optimizes the RCS, ultimately constructing and validating a four-factor structural model tailored to the local inquiry context of Chinese special education teachers. This model encompasses four core dimensions: Conceptual competence, Methodological competence, Data analysis and Interpretation, and Writing Technical Competency.

The findings of this study underscore the necessity of localizing a general RC framework within the context of special education practice, thereby providing robust theoretical support and empirical evidence for the development of a RC assessment system for special education teachers. The localized RCS serves as a standardized measurement tool, grounded in a comprehensive theoretical foundation, stable structure, and empirical validation for assessing the RC of Chinese special education teachers. This scale not only accurately diagnoses the competence gaps and training needs of special education teachers but also establishes a reliable measurement foundation for promoting evidence-based practice (EBP) in special education, optimizing school-based research and training mechanisms, and facilitating related academic research.

## Data Availability

The raw data supporting the conclusions of this article will be made available by the authors, without undue reservation.
